# Prevalence, withdrawal symptoms and associated factors of khat chewing among students at Jimma University in Ethiopia

**DOI:** 10.1186/s12888-017-1284-4

**Published:** 2017-04-17

**Authors:** Tilahun Abdeta, Daniel Tolessa, Kristina Adorjan, Mubarek Abera

**Affiliations:** 1 Department of Psychiatry, School of Nursing and Midwifery, College of Medical and Health Sciences, Haramaya University, Harar, Ethiopia; 2 Department of Psychiatry, Medical College, Adama, Ethiopia; 3 Department of Psychiatry and Psychotherapy, Ludwig-Maximilians-University, Munich, Germany; 4 Institute of Psychiatric Phenomics and Genomics, Munich, Germany; 5 Center for International Health, Ludwig-Maximilians-University, Munich, Germany; 6 Department of Psychiatry, College of Health Sciences, Jimma University, Jimma, Ethiopia

**Keywords:** Prevalence, Khat use, Withdrawal symptoms, Student, Jimma University

## Abstract

**Background:**

Recently, khat chewing has become a common practice among high school, college, and university students. Regular khat chewing is thought to be a predisposing factor for different physical and mental health problems. It can lead to absenteeism from work and classes. In Ethiopia, to our knowledge no published study has investigated khat withdrawal symptoms. Therefore, this study was conducted to determine the prevalence, withdrawal symptoms, and associated factors of khat chewing among regular undergraduate students on the main campus of Jimma University in Ethiopia.

**Methods:**

The institution-based, cross-sectional study was conducted in January 2016. Data were collected from 651 main campus regular undergraduate students with a structured, self-administered questionnaire, entered into Epidata 3.1 and exported to SPSS version 20 for Windows. Bivariate and multivariate logistic regressions were used to explore associations and identify variables independently associated with khat chewing.

**Results:**

The study found that the lifetime and current prevalence of khat chewing among students were 26.3% (95% CI: 24.3, 28.3) and 23.9% (95% CI: 21.94, 25.86), respectively. About 25.7% of students started chewing after joining university, and 60.5% of these students started during their first year. The main reason given for starting khat chewing was for study purposes (54.6%), followed by socialization purposes (42.3%). Among current khat chewers, 72.9% reported that they had chewed khat for 1 year or more and 68.2% reported that they had experienced various withdrawal symptoms. The most frequently reported withdrawal symptoms were feeling depressed, craving, and feeling fatigued. Being male, attending a place of worship daily/2–3 times per week, cannabis use, smoking cigarettes, and having family members currently chewing khat were independently associated with khat chewing.

**Conclusions:**

This study found that large numbers of university students were currently chewing khat. In this study withdrawal symptoms and factors that significantly affect khat chewing were identified. Besides it gave new ideas regarding khat withdrawal symptoms in Ethiopia. It serves as a critical role of providing information to form rational foundation for public health policy, prevention and planning to bring change in contributing factors for Khat chewing. The finding will be serving as base line information for further study.

## Background

Substance abuse, including khat chewing, is as old as the history of mankind. Khat (*Catha edulis*) is a large green shrub that grows at high altitudes (between 1500 and 2000 m above sea level) in the region extending from eastern to southern Africa and in the Arabian Peninsula [1, 2]. The plant is known by different names in different countries: “chat” in Ethiopia, “qat” in Yemen, “mirra” in Kenya, and “qaadorjaad” in Somalia; however, “khat” is the name commonly used in the literature [3].

The origin of khat is not clear, but it is generally agreed that khat is native to Ethiopia (Hararghe zone) and was first used there [4]. Fresh khat leaves contain more than 40 chemicals that vary depending on where it was grown, type variation, etc [5]. However, most of the stimulant effect of khat is thought to come from the chemicals cathinone, cathine, and norephedrine, all of which stimulate the central nervous system in a similar way to amphetamine. Cathinone has been reported to be 7–10 times more potent than cathine [6, 7]. Cathinone plasma levels peak 1.5–3.5 h after khat chewing [8].

Recently, khat chewing has become a common practice among high school, college, and university students for recreational purposes and because they believe that it increases their academic performance [9, 10]. The World Health Organization (WHO) has classified khat as a drug of abuse that can produce mild to moderate psychological dependence [11–14]. According to the WHO, chronic khat use can result in a variety of physical illnesses, including urinary retention, impotence, oral cancer, dental caries, chronic gastritis, hemorrhoids, paralytic ileus, liver cirrhosis, hypertension, and blurred vision. Besides, it can cause psychotic and suicidal depressive reactions, mania and have effects on the central nervous system, e.g. dizziness, impaired cognitive functioning, fine tremor, insomnia, and headaches. Khat chewers have a significantly higher mortality rate due to chronic illnesses such as heart disease and stroke than non-khat chewers, and khat chewing among youths can increase the risk of contracting human immunodeficiency virus (HIV) and other sexually transmitted diseases [15–22].

Khat chewers spend long hours chewing and then recovering from chewing, which can lead to a loss of work hours and absenteeism from work and classes and potentially result in a decrease in overall national economic productivity and poor academic performance among students [23–26].

The available literature is inconsistent regarding the presence or absence of khat withdrawal symptoms. One study on the pharmacological and medical aspects of khat and its social use in Yemen found that when chronic khat chewers stop chewing, they develop withdrawal symptoms such as feeling hot in their legs, nightmares, mild depression, slight tremor, lethargy, a desire to chew khat, and so on [27]. However, another study on substance abuse in outpatients attending rural and urban health centers in Kenya found no signs of khat dependency or withdrawal symptoms [28]. No research is available on the prevalence, withdrawal symptoms, and associated factors of khat chewing in our study area, and we found no study on khat withdrawal symptoms in other regions of Ethiopia. Therefore, the objective of this study was to determine the prevalence, withdrawal symptoms, and associated factors of khat chewing among regular undergraduate students of the main campus at Jimma University, Ethiopia, to provide additional information on the prevalence of khat chewing and khat withdrawal symptoms.

## Methods

### Study area and period

This study was conducted in January 2016 among 651 regular undergraduate students of the main campus at Jimma University, Ethiopia. Jimma University is located in Oromia regional state, 352 km from Addis Ababa, in the southwest of the country and is one of the public higher education institutions in Ethiopia. The university currently has four campuses: (I) Main Campus; (II) College of Agriculture and Veterinary Medicine; (III) Kitofurdisa campus (Institute of Technology/IOT); and (IV) Business and Economics College. Our study was conducted at the Main Campus, which has three colleges (College of Health Sciences, College of Natural Science, and College of Social Science and Law). The reason why we chose main campus is; it has relatively many students of different colleges and many departments under each college. But the rest campuses have very specific colleges which could affect our results especially the prevalence of chewing khat. The main campus has a total of 6304 regular undergraduate students, 4599 of whom are male and 1705 female.

### Study design

Institution based cross-sectional study design was facilitated.

### Source population

All regular undergraduate students registered for the 2016 academic year in main campus, Jimma University were considered as source population.

### Study population

All selected regular undergraduate students registered for 2016 academic year in main campus, Jimma University were considered as a study population.

### Inclusion criteria

All regular undergraduate students registered from 1st to 6th year and attending their education during the study period in main campus, Jimma University were included in the study.

### Sample size determination

From the results of a previous study done on the subject matter among Ethiopian university students we took population proportion 27.9% [29]. Using the formula for the sample size of single population proportion:

$$ \mathrm{ni}=\frac{{\left( za/2\right)}^2 p\left(1- p\right)}{{\mathrm{d}}^2} $$

Where

ni = initial sample size

*a* = confidence interval (95%)

p = proportion of Khat chewing: 27.9%,

d = is the margin of sampling error tolerated (5%)

$$ \mathrm{ni}=\frac{(1.92)^2.0.279\left(1-0.279\right)}{(0.05)2}=\frac{3.8416\times 0.279\times 0.721}{0.0025}=310 $$

Since the total number of regular students in the main campus is 6304 which is less than 10,000, Using finite population correction formula the final sample size was;

$$ {\mathrm{n}}_{\mathrm{f}}=\frac{ni}{1+\left( ni/ N\right)} $$

where,

nf = final sample size

ni = initial sample size calculated above (310)

N = total number of undergraduate regular students.

$$ {\mathrm{n}}_{\mathrm{f}}=\frac{310}{1+\left(310/6304\right)}=296 $$

Since we had employed a multistage sampling technique we multiplied the sample size by two in order to correct bias that was introduced in the sampling design. Therefore, the final sample size became 296 × 2 = 592. Again this is a self-administered study as a result we considered a 10% non-response rate. So, by adding 10% non-response rate, the final sample size was 651.

### Sampling procedure

**Fig. 1:**
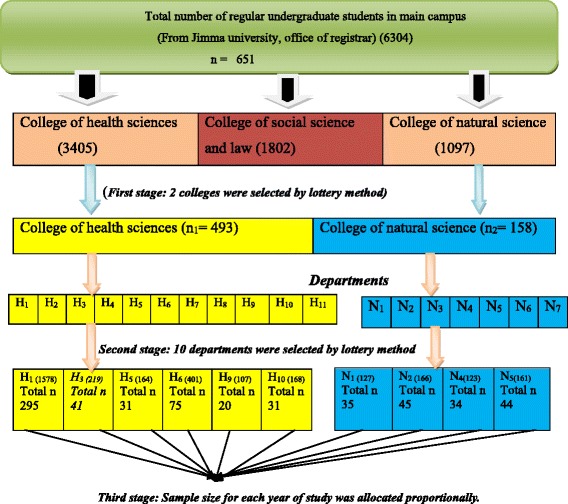
The schematic presentation of the sampling procedure employed among undergraduate students, Jimma University, Ethiopia, 2016

The study was conducted using a multistage sampling technique. First stage was formed using colleges and two out of three colleges were selected to increase the representativeness through lottery method. In the second stage again 10 departments in the selected colleges were proportionally allocated. Sample size for each department and year of study was allocated according to proportion to the number of students in the specific department and year of study by using the following proportional allocation formula, and each respondent was selected by a simple random sampling technique (see Fig. 1).

Proportional allocation formula: ${\mathrm{n}}_{\mathrm{x}}=\frac{n. Nx}{NT}$.

Where:

nx = sample from department x

Nx = total number of student in department x

NT = total number of students in the selected collages and

n = total sample size

### Study variables

#### Dependent variables

- ✓ History of current khat chewing

#### Independent variables

- **➢** Socio-demographic factors
  Age, Sex, Marital status, Ethnicity, Religion, Frequency of attending a place of worship, Monthly pocket money, Residence before, Current and previous Living condition, Family’s main source of income, Families chewing habit.
- **➢** Substance use other than khat use
  Alcohol use, Cigarette smoking, Cannabis use, Shisha and other substances use.
- **➢** School related factors
  Cumulative grade point, colleges, Year of study.

### Data collection procedures

A self-administered, structured questionnaire adapted from the literature was used. The questionnaire had three parts: (I) socio-demographic information; (II) khat chewing habits and substance use other than khat; and (III) checklist to assess khat withdrawal symptoms. The questionnaire was translated into both Amharic and Afan Oromo, languages that most students could understand. Both versions were then back-translated into English by an independent translator. Finally, the questionnaire was administered in both Amharic and Afan Oromo. Six supervisors (three clinical nurses [BSc] and three psychiatric nurses [BSc]) and the principal investigator closely supervised the overall data collection. Data collectors were nine psychiatric nurses (four MSc and five BSc), and one BSc clinical nurse.

### Data quality management

The questionnaire was pre-tested by data collectors on 33 students from the campus of College of Agriculture and Veterinary Medicine 1 week before the start of data collection for the main study. Data collectors and Supervisors were given 1 day of training. Collected data were checked for completeness, coded, entered into Epidata 3.1 and then exported to SPSS version 20 for Windows for statistical analysis.

### Data processing, analysis and interpretation

After all necessary data obtained, data were checked manually for completeness and questionnaires with incomplete data were excluded. Data were edited, coded and entered in to Epidata version3.1 then exported and analyzed by SPSS version 20 for windows. Descriptive statistics such as measures of central tendency, standard deviations, and frequencies including cross tabulation were used to summarize data. Bivariate and multivariate logistic regressions were used to explore the presence of associations and identify independent predictors of khat chewing. Variables with a *p*-value <0.25 in the bivariate analysis were entered into multivariate logistic regressions in order to control for potential confounders and calculate adjusted odds ratios with a 95% confidence interval (95% CI). Variables with *p* < 0.05 in the multivariate logistic regressions were considered as significantly associated with khat chewing.

### Ethical consideration

The study was approved by the ethical review board of the College of Health Sciences, Jimma University. Immediately before the questionnaire was distributed, written informed consent was obtained from the study participants after they had been given a clear explanation of the study objectives. The collected data were kept confidential. The participants were informed about their right to refuse to participate and to ask anything about the study. The names of the study participants were not written in order to keep their privacy. Beside the study was conducted using self administered questionnaire which ensures participants’ privacy and encourages them to give their ideas freely. Since the study was conducted by self administered questionnaire and due to privacy issue we could not find individuals with problem (khat chewing). However since the prevalence of current khat chewing in this study was relatively high; we had given mass-education for all under graduate students of main campus with suggesting the availability of help/treatment at psychiatry clinic of Jimma University specialized hospital and Students with the problem can be evaluated and get appropriate treatment in the hospital.

### Operational definitions

Lifetime prevalence of khat chewing: Defined as the proportion of students who had ever used khat at least once in their lifetime [30].

Current prevalence of khat chewing: Defined as the proportion of students who used khat at least once during the last 1 month preceding the study [31].

Khat withdrawal symptoms: The usually unpleasant set of physical or psychological symptoms experienced due to the cessation of (or reduction in) khat use during the last 1 month preceding the study [32].

Craving: Feeling desire to chew khat again and again [32].

## Results

### Socio-demographic characteristics of respondents

Of 651 students provided with self-administered questionnaires, 619 completed and returned them (response rate: 95.1%); 75.0% of the respondents were males and 25.0% females. The mean (SD) age of participants was 21.89 (2.30) years and the majority (97.1%) were single. Large proportions (39.7%) of the respondents were Orthodox Christians, followed by Muslims (29.6%). The majority of respondents (62.2%) were of Oromo ethnicity, followed by Amhara (20.5%). Most respondents (61.7%) were originally from an urban area, had previously been living with their family (94.0%) and were currently living in a dormitory (93.5%). The main source of income of the respondents’ families was agriculture (40.7%), followed by employment in governmental institutions (29.1%). Large proportion of the students (91.1%) received some monthly pocket money. The 77.9% of the students were from Health Sciences College, and students in the first year of study of their respective department accounted for the largest proportion (24.6%), followed by third-year students (23.9%). Apart from the first-year students (24.6%), all students (75.4%) reported their cumulative Grade Point Average (cGPA); large proportion of them (51%) had a cGPA <3.25 (see Table 1).

**Table 1:** Socio-demographic characteristics of undergraduate students at Jimma University, Ethiopia, 2016

Socio-demographic characteristics	Frequency (n)	Percent (%)
Sex (*n* = 619)		
Male	464	75.0
Female	155	25.0
Age (*n* = 619)		
< 20	200	32.3
20–24	326	52.7
25 and above	93	15.0
Marital status (*n* = 619)		
Single	601	97.1
Married	18	2.9
Religion (*n* = 619)		
Orthodox Christian	246	39.7
Muslim	183	29.6
Protestant	157	25.4
Other^a^	33	5.4
Frequency of attending a place of worship (*n* = 619)		
Daily	257	41.5
2–3 times per week	188	30.4
Weekly	94	15.2
Less than weekly	37	6.0
Never	43	6.95
Ethnicity (*n* = 619)		
Oromo	385	62.2
Amhara	127	20.5
Guraghe	22	3.6
Tigre	13	2.1
Wolayita	23	3.7
Other^b^	49	7.9
Area of residence before joining university (*n* = 619)		
Urban	382	61.7
Rural	237	38.3
Living situation before joining university (*n* = 619)		
Living alone	29	4.7
Living with family	582	94.0
Other^c^	8	1.3
Current living situation (*n* = 619)		
Dormitory	579	93.5
Rented accommodation	28	4.5
Other^d^	12	2
Family’s main source of income (*n* = 619)		
Agriculture	252	40.7
Government job	180	29.1
Trade	142	22.9
NGO/private firm work	32	5.2
Other^e^	13	2.1
Average monthly pocket money (Ethiopian birr) (*n* = 564)		
1–100	26	4.2
101–299	96	15.5
300–499	165	26.7
500–999	188	30.4
1000 and above	89	14.4
College		
College of Health Sciences	482	77.9
College of Natural Sciences	137	22.1
Year of study (*n* = 619)		
First year	152	24.6
Second year	141	22.8
Third year	148	23.9
Fourth year and above	178	28.7
Cumulative grade point average (cGPA) (*n* = 467)		
< 3.25/ less than distinction level	238	51
≥ 3.25/ distinction level	229	49

^a^Catholic, Hawariyat, or traditional

^b^Sidama, Kaffa, Dawro, or Somali

^c^Living with relatives or in own home with husband or wife

^d^Living with relatives or family

^e^Own business

### Khat chewing practice

The lifetime and current prevalence of khat chewing among students were (26.3%; 95% CI: 24.3, 28.3) and (23.9%; 95% CI: 21.94, 25.86), respectively. The majority of current khat chewers (66.9%) reported that they started khat chewing after the age of 15 years. The mean (SD) age for starting khat chewing was 17.2 (3.2).

Among the current khat chewers, 36.5% chewed khat 2–3 times a week, 35.1% daily, 25.7% weekly, and 2.7% occasionally. Majority of them (54.1%) spent >25 Ethiopian Birr (ETB) on khat for each session, and the others 16–25 ETB (27.7%) and ≤15 ETB (18.2%). The mean duration of a khat chewing session was 2–4 h for 41.2% of the current khat chewers, <2 h for 29.7% and >4 h for 29.1%.

### Reasons reported by respondents for starting khat chewing

**Fig. 2:**
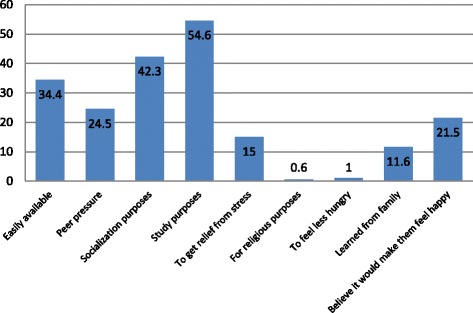
Reasons for starting khat chewing among undergraduate students at Jimma University, Ethiopia, 2016

A total of 74.3% of current chewers started chewing khat before joining university. The remainder (25.7%) started chewing after joining university, mostly in their first year (60.5%). The main reason given for starting khat chewing was for study purposes (54.6%), followed by socialization purposes (42.3%) (See Fig. 2).

### Khat withdrawal symptoms

**Fig. 3:**
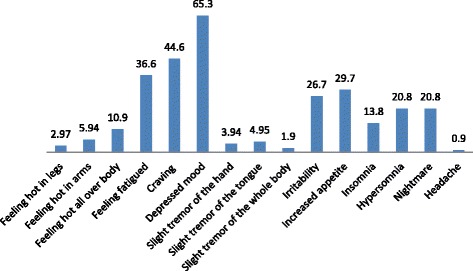
Withdrawal symptoms reported among undergraduate students at Jimma University, Ethiopia, 2016

Among current khat chewers, 72.9% reported that they had chewed khat for 1 year or more and the majority (68.2%) of them reported experiencing different withdrawal symptoms when they stopped chewing khat or reduced the amount of khat they chewed. The most frequently reported withdrawal symptoms were feeling depressed (65.3%), craving (44.6%), feeling fatigued (36.6%), increased appetite (29.7%), irritability (26.7%), hypersomnia (20.8%) and nightmares (20.8%). Besides these symptoms, insomnia (13.8%), feeling hot all over (10.9%), feeling hot in arms (5.9%), slight tremor of the tongue (4.95%), slight tremor of the hand (3.94%), feeling hot in legs (2.97%), slight tremor of the whole body (1.9%) and headache (0.9%) were also reported. (See Fig. 3). Among those who reported withdrawal symptoms, 55.4% usually felt the symptoms for more than ten hours, 7.9% for 6–10 h, 23.8% for 1–5 h, and 12.9% for <1 h. Also, a large proportion of them (66.3%) reported that the withdrawal symptoms cause impairment in social, occupational, or other important areas of their functioning. The majority of those who reported having withdrawal symptoms (52.5%) use different measures to get relief: 34% use coca or coffee, 28.3% chew khat, 20.75% sleep, 11.3% do sport or watch a movie, 3.7% take a shower and 1.9% use candy for a long time.

### Factors independently associated with current khat chewing

To determine the association between dependent and independent variables, we performed bivariate and multivariate logistic regression analyses. The multivariate logistic regression analysis found that male students had 2.3 times higher odds of chewing khat than females (AOR2.3, 95% CI: 1.16, 4.6).

Irrespective of their religion, students who were attending a place of worship daily or 2–3 times per week were 90% less likely to chew khat than students who never attended a place of worship during the last month (AOR 0.1, 95% CI: 0.03, 0.29). Students those who have family members currently chewing khat had 15.6 times higher odds of chewing khat than those who have no family members currently chewing khat (AOR 15.6, 95% CI: 8.3, 29.5). Students who had used cannabis at least once during the last month had more than four times higher odds of chewing khat than those who did not use it during the last month (AOR 4.17, 95% CI: 1.6, 11.2). Students those who had not smoked cigarettes during the last month were 55% less likely to chew khat than those who had smoked cigarettes at least once during the last month (AOR 0.45, 95% CI: 0.3, 0.7).

Age, marital status, ethnicity, family’s main source of income, and current drinking alcohol were statistically significant in the binary logistic regression analysis but not significant when adjusted for other variables in the final model (See Tables 2 and 3).

**Table 2:** Socio-demographic factors independently associated with current khat chewing among undergraduate students, Jimma University, Ethiopia, 2016

Variables	Current khat chewing		*P*-value	AOR (95% CI)
	Yes	No		
Age				
< 20	38 (19)	162 (81)	0.5	0.75 [0.3**–**1.8]
20–24	78 (23.9)	248 (76.1)	Reference	Reference
≥ 25	32 (34.4)	61 (65.6)	0.49	0.76 [0.35**–**1.67]
Sex				
Male	125 (26.9)	339 (73.1)	0.02	2.3 [1.16**–**4.6]
Female	23 (14.8)	132 (85.2)	Reference	Reference
Marital status				
Single	139 (23.1)	462 (76.9)	Reference	Reference
Married	9 (50)	9 (50)	0.44	0.49 [0.08**–**3.0]
Ethnicity				
Oromo	112 (29.1)	273 (70.9)	Reference	Reference
Amhara	15 (11.8)	112 (88.2)	0.06	2.1 [0.96**–**4.5]
Others^a^	21 (19.6)	86 (80.4)	0.3	0.56 [0.18**–**1.7]
Religion				
Muslim	91(49.7)	92 (50.3)	Reference	Reference
Orthodox	41(16.7)	205 (83.3)	0.001	6.7 [2.8**–**16.3]
Others^b^	16 (8.4)	174 (91.6)	0.001	4.0 [1.76**–**9.37]
Frequency of attending a place of worship				
Daily/2–3 times per week	96 (21.6)	349 (78.4)	0.001	0.1 [0.03–0.29]
Weekly	28 (29.8)	66 (70.2)	0.3	0.55 [0.17**–**1.7]
Less than weekly	7 (17.1)	34 (82.9)	0.13	0.19 [0.04**–**0.84]
Never	17 (43.6)	22 (56.4)	Reference	Reference
Previous living condition				
Living Alone	4 (13.8)	25 (86.2)	Reference	Reference
With family	140 (24.1)	442 (75.9)	0.13	0.15 [0.14–1.73]
Others^c^	4 (50)	4 (50)	0.29	0.34 [0.05**–**2.5]
Current living condition				
Dormitory	125 (21.6)	454 (78.4)	Reference	Reference
In rented home	21 (75)	7 (25)	0.5	0.38 [0.02**–**6.15]
Others^d^	2 (16.7)	10 (83.3)	0.4	3.56 [0.18**–**71.5]
Monthly pocket money (Ethiopian Birr)				
≤ 100	3 (11.5)	23 (88.5)	Reference	Reference
101–299	15 (15.6)	81 (84.4)	0.95	0.95 [0.18**–**4.85]
300–499	35 (21.2)	130 (78.8)	0.95	0.97 [0.33**–**2.85]
500–999	61 (32.4)	127 (67.6)	0.33	1.58 [0.63–3.97]
≥ 1000	27 (30.3)	62 (69.7)	0.4	1.4 [0.61**–**3.37]
Family’s main source of income				
Agriculture	54 (21.4)	198 (78.6)	Reference	Reference
Trade	48 (33.8)	94 (66.2)	0.61	1.8[0.16**–**21.75]
Government job	33 (18.3)	147 (81.7)	0.58	1.9 [0.17**–**23.4]
NGO/private firm work	10 (31.25)	22 (68.75)	0.73	1.53[0.13**–**17.45]
Others^e^	3 (23.1)	10 (76.9)	0.37	3.4 [0.24**–**48.3]
Family members currently chew khat				
Yes	105 (63.6)	60 (36.4)	0.001	15.6 [8.3–29.5]
No	43 (9.5)	411 (90.5)	Reference	Reference

^a^Guraghe, Tigre, Wolayita, Sidama, Kaffa, Dawro, or Somali

^b^Protestant, Catholic, Hawariyat, or traditional

^c^Living with relatives or in own home with husband or wife

^d^Living with relatives or family

^e^Ownbusiness

**Table 3:** Substance use other than khat and School related factors independently associated with current khat chewing among undergraduate students, Jimma University, 2016

Variables	Current khat chewing		*P*-value	AOR (95% CI)
	Yes	No		
Cumulative grade point average (cGPA)				
< 3.25 (less than distinction level)	67 (28.2)	171 (71.8)	Reference	Reference
≥ 3.25 (distinction level)	50 (21.8)	179 (78.2)	0.38	0.82 [0.53**–**1.27]
Alcohol used at least once during the last 1 month				
No	82 (19.2)	345 (80.8)	Reference	Reference
Yes	66 (34.4)	126 (65.6)	0.53	1.15 [0.7**–**1.8]
Smoking cigarettes at least once during the last 1 month				
No	89 (18.5)	392 (81.5)	0.001	0.45 [0.3**–**0.7]
Yes	59 (42.8)	79 (57.2)	Reference	Reference
Ganja/cannabis used at least once during the last 1 month				
No	134 (22.4)	463 (77.6)	Reference	Reference
Yes	14 (63.6)	8 (36.4)	0.004	4.17 [1.6**–**11.2]
Shisha used at least once during the last 1 month				
No	129 (24)	408 (76)	Reference	Reference
Yes	19 (23.2)	63 (76.8)	0.9	1.02 [0.51**–**2.06]

## Discussion

This study assessed the prevalence, withdrawal symptoms, and associated factors of khat chewing.

The current prevalence of khat chewing (23.9%) found in this study is in line with that found among students at Jazan University, Saudi Arabia (23.1%) [33] And undergraduate students at Haramaya University, Ethiopia (23.6%) [34]. However, the prevalence is higher than that found at Addis Ababa University and Gondar University and at four other colleges in North West Ethiopia [10, 35, 36]. The possible reasons for the different rates could be differences in the study settings, such as access to khat, and factors outside the university environment, for example in Jimma town chewing khat is more common and is normalized by the community [37]. The prevalence in this study was also lower than that in a study conducted among students at Axum University in April 2012 (27.9%) [29]; this difference could be due to different sample sizes.

In our study, a large proportion of current khat chewers (74.3%) reported that they started chewing khat before joining university. Among those who started after joining university (25.7%), the majority (60.5%) started when they were first-year students. This result is supported by previous findings at Gondar University and four other colleges in North West Ethiopia [15, 36]. A possible explanation for the higher rate among first-year students may be a maladaptive response to stressors such as the new environment, academic stress, separation from their family, and the contents of learning. In agreement with these explanations, in this study the main reason given for starting khat chewing was for study purposes (54.6%), followed by socialization purposes (42.3%). All these statements are important indicators for interventions aimed at decreasing the prevalence of khat chewing among undergraduate students at university.

This study found that 36.5% of current khat chewers chew khat 2–3 times a week, 54.1% chew khat costing >25 ETB per chewing session, and 29.1% spend >4 h per chewing session. These findings were comparable to those of other studies among university students at Jazan University, Saudi Arabia [33], and college students in Bahir Dar town [38], Ethiopia, and indicate that students spend a lot of money and time on khat chewing. This use of resources may have an impact on social and economic aspects of the students’ lives and their health and academic performance. Furthermore, if students have no money to buy khat they could be tempted to engage in criminal activities.

To our knowledge this study is the first in Ethiopia to investigate khat withdrawal symptoms. The findings of the few available studies regarding the presence or absence of khat withdrawal symptoms are inconsistent. The findings of the current study are in line with those of one previous study finding in Yemen, which showed that a chronic khat chewer who stops chewing the leaf feels hot, especially in legs, and lethargic, has a desire to chew khat and has nightmares, mild depression, and a slight tremor [27]. This finding is comparable also to DSM-5 stimulant withdrawal diagnostic criterion “B,” which specifies that dysphoric mood and two or more of the following physiological changes (fatigue, unpleasant dreams, insomnia or hypersomnia, increased appetite, and psychomotor retardation or agitation) should occur within a few hours to several days [32]. On the other hand, this finding was inconsistent with that of a study in Kenya, which found no signs of khat dependency or withdrawal symptoms [28]. In the present study, the mean duration of withdrawal symptoms was 23.3 h. More than half (55.4%) of the study participants who had withdrawal symptoms reported that it lasted for more than ten hours. A large proportion of those with withdrawal symptoms (66.3%) reported that the symptoms caused impairment in social, occupational, or other important areas of their functioning. Just over half (52.5%) of them reported they took different measures to get relief from the withdrawal symptoms: 34% use coca or coffee, 28.3% chew khat again, 20.75% sleep, 11.3% do sport or watch a movie, 3.7% take a shower, and 1.9% use candy for a long time. These findings are important indications of ways to improve health education for students and also show that students spend time and money to get relief from withdrawal symptoms.

We found that the following independent variables are significantly associated with khat chewing: male gender, attending a place of worship daily/2–3 times per week, current use of ganja/cannabis, current smoking cigarettes and having family members who are current khat chewers.

In our study, male students had 2.3 higher odds of chewing khat than female students [AOR 2.3, 95% CI: 1.16, 4.6]. This finding is supported by similar findings from studies among Ethiopian students at Axum University and Haramaya University and Ethiopian college students in Bahir Dar town [29, 34, 38]. The difference between males and females could be due to cultural restrictions, because in Ethiopia females are less exposed to khat chewing than males [38].

Irrespective of their religion, students who were attending a place of worship daily or 2–3 times per week were 90% less likely to chew khat than students who never attended a place of worship during the last month (AOR 0.1, 95% CI: 0.03, 0.29). This result is in line with that of a previous study at Gondar University [35]. A possible explanation for this finding could be that substance use is not supported by religious teaching [15].

Students those who have family members currently chewing khat had 15.6 times higher odds of chewing khat than those who have no family members currently chewing khat (AOR 15.6, 95% CI: 8.3, 29.5). This finding is in agreement with the findings of previous studies in college students in Bahir Dar Town [38] and students at Axum University in Ethiopia [29] and Jazan University in Saudi Arabia [33]. Khat chewing could be associated with chewing among family members for a number of reasons: the family might have a common protective factor that serves all family members, such as family policy, religion, etc.; and khat chewing might be less socially acceptable in families that do not chew khat.

Students who had used cannabis at least once during the last month had more than four times higher odds of chewing khat than those who did not use it during the last month (AOR 4.17, 95% CI: 1.6, 11.2). This finding shows that poly-substance use behavior can be found among students with a khat chewing habit [15].

Students those who had not smoked cigarettes during the last month were 55% less likely to chew khat than those who had smoked cigarettes at least once during the last month (AOR 0.45, 95% CI: 0.3, 0.7). This finding is in agreement with study conducted among university students in Northwest Ethiopia [35].

This study has some limitations. First, the cross-sectional study design does not determine a cause and effect relationship (the chicken and egg dilemma). Second, self-administered data might increase the social desirability bias and non response rate, which can underestimate the prevalence of khat chewing. Despite these limitations, the study has several strengths, including the following: (I) the response rate was relatively high and the sample size was relatively large; (II) the sampling procedure and analysis methods were appropriate for the study; (III) the study investigated withdrawal symptoms for the first time in Ethiopia.

## Conclusions

To conclude, this study found that large numbers of university students chew khat. The study gave new ideas regarding khat withdrawal symptoms in Ethiopia. The most frequently reported withdrawal symptoms were feeling depressed, craving, and feeling fatigued. Factors those were significantly associated with khat chewing were male gender, attending a place of worship daily/2–3 times per week, currently using ganja/cannabis, current smoking cigarettes and having family members who chew khat. The study findings serve as a critical role of providing information to form rational foundation for public health policy, prevention and planning to bring change in contributing factors for Khat chewing. The finding will be serving as base line information for further study.

## Abbreviations

AOR: Adjusted odds ratio
CGPA: Cumulative grade point average
COR: Crude Odds Ratio
ETB: Ethiopian Birr
IOT: Institute of Technology
NGO: Non Government Organization
SPSS: Statistical Package for Social Science
WHO: World Health Organization
